# Survival Study: International Multicentric Pancreatic Left Resections (SIMPLR-2): Does Surgical Approach Matter for Recurrence-Free Survival and Overall Survival?

**DOI:** 10.3390/cancers17162659

**Published:** 2025-08-15

**Authors:** Sara Al-Madhi, Mohammad Abu Hilal, Sara Acciuffi, Mirhasan Rahimli, Seong Jeong, Karol Rawicz-Pruszyński, Marc-Anthony Chouillard, Nouredin Messaoudi, Elie Chouillard, Ibrahim Dagher, Roland S. Croner, Andrew A. Gumbs

**Affiliations:** 1 Department of General, Visceral, Vascular and Transplantation Surgery, University of Magdeburg, 39120 Magdeburg, Germany; sara.al-madhi@med.ovgu.de (S.A.-M.); sara.acciuffi@med.ovgu.de (S.A.); mirhasan.rahimli@med.ovgu.de (M.R.); seong.jeong@med.ovgu.de (S.J.); roland.croner@med.ovgu.de (R.S.C.); 2 Department of Surgery, University Hospital Southampton NHS Foundation Trust, Southhampton SO16 6YD, UK; abuhilal9@gmail.com; 3 Department of Surgical Oncology, Medical University of Lublin, Radziwiłłowska 13 St., 20-080 Lublin, Poland; krpruszynski@gmail.com; 4 Hepatobilipancreatic Surgery, Assistance Publique des Hôpitaux de Paris, 75019 Paris, France; marcanthony.chouillard@protonmail.com; 5 Department of Surgery, Vrije Universiteit Brussel (VUB), Universitair Ziekenhuis Brussel (UZ Brussel) and Europe Hospitals, 1090 Brussels, Belgium; nouredinmessaoudi@gmail.com; 6 American Hospital of Paris, 92200 Neuilly-sur-Seine, France; elie.chouillard@ahparis.org; 7 Hôpital Antoine Béclère, Assistance Publique-Hôpitaux de Paris, 157 Rue de la Porte de Trivaux, 92140 Clamart, France; ibrahim.dagher@aphp.fr

**Keywords:** pancreatic ductal adenocarcinoma, left pancreatectomy, minimally invasive surgery, robotic surgery, laparoscopic pancreatectomy, overall and recurrence-free survival

## Abstract

**Introduction**: SIMPLR-2 is a follow-up to the international, multicenter SIMPLR-1 study evaluating long-term oncological outcomes after open (OLP), laparoscopic (LLP), and robotic (RLP) left pancreatectomy for pancreatic ductal adenocarcinoma (PDAC). **Methods**: A retrospective analysis of 71 PDAC patients from three high-volume centers was performed. Surgical approaches were stratified into open, laparoscopic, and robotic. The primary endpoint was overall survival (OS); recurrence-free survival (RFS) was also assessed. Kaplan–Meier and Cox regression analyses were used, including propensity score adjustment. **Results**: Median OS was 11.0 months for OLP and 24.0 months for minimally invasive approaches (*p* = 0.169). Median RFS was 6 vs. 15 months, respectively (*p* = 0.258). No significant differences were found between surgical approaches. **Conclusions**: Although longer survival was observed in minimally invasive groups, differences were not statistically significant. Larger prospective trials are needed to clarify the long-term oncologic impact of a surgical approach in PDAC.

## Simple Summary

This study is a continuation of the international multicenter cohort, SIMPLR-1, conducted across three high-volume centers, and investigates long-term outcomes after a minimally invasive left pancreatectomy in patients with pancreatic malignancies. Surgical approaches—open, laparoscopic, and robotic—were compared with regard to overall and recurrence-free survival. While previous results from SIMPLR-1 suggested short-term advantages of minimally invasive techniques over open surgery, SIMPLR-2 did not reveal a clear survival benefit for any specific approach. Further randomized and sufficiently powered trials are needed to determine whether minimally invasive strategies offer long-term oncologic advantages.

## 1. Introduction

Minimally invasive left pancreatectomy (MILP) for pancreatic tumors has progressively gained acceptance since it was first performed laparoscopically by Cuschieri 1994 [1] and Melvin et al., who performed the first robotic left pancreatectomy (RLP) in 2003 [2]. Nowadays, it is considered to be a standard safe practice in many high-volume, expert centers for both benign and selected malignant lesions [3,4]. Pancreatic ductal adenocarcinoma (PDAC) accounts for the majority of pancreatic cancers, with 20–25% located in on the left side of the pancreas [5,6]. Most of these tumors are asymptomatic which makes early detection challenging, with over 50% presenting with metastasis at the time of diagnosis [5,7,8]. Only less than 10% of all patients represent with resectable disease [9]. Furthermore, the Surveillance, Epidemiology, and End result (SEER) registry has revealed that patients with PDAC located in the body and tail of the pancreas (21.1% of the total 67,878 patients analyzed) are associated with a worse prognosis compared to those with PDAC located in the head [5,8]. This makes distal PDAC have a lower relative 5-year survival, with only about 9% overall survival [10,11]. Surgical resection followed by adjuvant chemotherapy remains the only curative treatment for PDAC, particularly in early-stages [7,11].

The decision regarding surgical approach—whether open, laparoscopic, or robotic—requires careful multidisciplinary evaluation. Van Ramshorst et al. reported in a recent international survey that MILP has gained more popularity, with 94.6% of surgeons preferring MILP over the OLP [4,12]. However, a laparoscopic approach is still more utilized due to the availability and the lower cost compared to the robotic approach [4,12]. According to recent reports, the robotic approach is expanding and is increasingly favored due to its technical advantages, enhancing dexterity, 3D high-resolution visualization, and tremor filtration, allowing surgeons to perform more technically challenging procedures [4,12,13]. Nevertheless, cost remains an issue for many healthcare providers [12,13]. However, in locally advanced pancreatic cancer with larger tumor and vascular involvement, particularly those tumors located in the pancreatic body, an open surgical approach is generally still preferred after neoadjuvant treatment, especially when vascular resection and reconstruction are anticipated [14,15].

Both laparoscopic left pancreatectomy (LLP) and robot-assisted left pancreatectomy are associated with a shorter hospital stay and faster recovery compared with open left pancreatectomy (OLP) [12,16]. The short-term clinical advantages of MILP have been demonstrated in three randomized controlled trials, confirming that MILP is a safe and feasible approach with regard to morbidity and mortality compared to OLP [9,17].

Despite the growing adoption of MILP, some surgeons are still concerned about the oncological safety in resectable PDAC [9,18]. Several retrospective cohort studies have shown no significant differences in the R0 resection rate and lymph node harvest between LLP, RLP, and OLP in terms of oncological quality and safety [9,15,19]. A recent cohort from two multicenter studies comparing LLP and RLP have shown lower conversion rates with a higher lymph node harvest, maintaining a similar R0 resection rate with a longer operative time in the RLP cohort [16,20]. A recent systemic review and meta-analysis of 21 studies involving 11,246 patients with distal PDAC showed comparable oncological safety [21]. Additionally, the DIPLOMA study, a large international multicenter propensity score-matched study, demonstrated non-oncological inferiority of MILP compared to OLP [9]. Furthermore, the study revealed a better scar satisfaction score among patients at one year of surgery in the minimally invasive group [9,22]. However, with regard to the long-term oncological outcomes among different surgical approaches, no studies have directly compared the oncological outcomes individually between the three surgical approaches LLP, RLP, and OLP.

The International Multicentric Pancreatic Left Resections (SIMPLR-1) was a propensity score-matched (PSM) study comparing the three surgical approaches, open left pancreatectomy (OLP), laparoscopic left pancreatectomy (LLP), or robotic left pancreatectomy (RLP), for patients with benign and malignant tumors and focused on the short-term outcomes of patients with tumors of the pancreatic body and tail. The laparoscopic technique demonstrates superior short-term advantages, including reduced hospital and ICU stays, as well as shorter operative times compared to both other techniques, without compromising oncologic outcomes [12]. Overall, no significant differences were observed among the three surgical approaches in terms of major morbidity, mortality, or oncological safety [12].

Designated as SIMPLR-2, this study aimed to determine whether differences exist in long-term outcomes, specifically overall survival (OS) and recurrence-free survival (RFS), between open, laparoscopic, and robotic left pancreatectomy, focusing only on patients with PDAC and excluding other tumors.

## 2. Materials and Methods

### 2.1. Study Design and Population

This retrospective, multicentric study analyzed patients from the SIMPLR-1 cohort who underwent a left pancreatectomy for pancreatic ductal adenocarcinoma (PDAC) between January 2016 and December 2020 at three high-volume centers. Previously used inclusion and exclusion criteria were reported in SMPLR-1.

**Figure 1:**
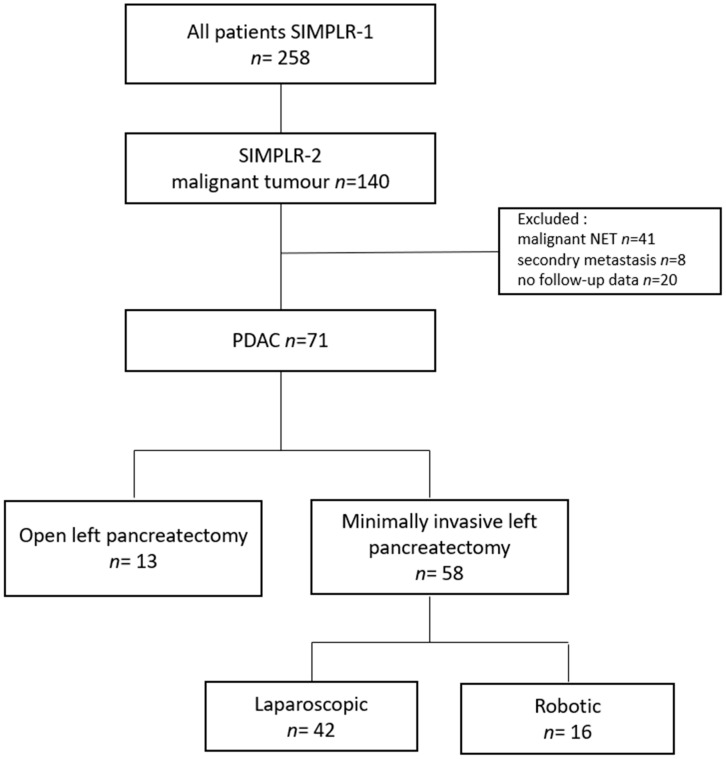
Flowchart of patient selection for the SIMPLR-2 study. From 258 patients who underwent left-sided pancreatectomy in SIMPLR-1, 140 had a malignant pathology. Only patients with PDAC and complete follow-up data were included in SIMPLR-2 (*n* = 71). Exclusion criteria were neuroendocrine tumors (NET), secondary metastases, and missing follow-up.

Of the 258 patients with left-sided pancreatic tumors enrolled in SIMPLR-1, 140 were diagnosed with a malignant pathology. For the SIMPLR-2 study, we included only patients with histologically confirmed pancreatic ductal adenocarcinoma (PDAC) and available long-term follow-up data. Patients with neuroendocrine tumors (*n* = 41), pancreatic metastases from other primary tumors (*n* = 8), or missing follow-up information (*n* = 20) were excluded. The final cohort included 71 patients with PDAC. All histopathological assessments were performed locally at the participating centers; no central pathology review was conducted (Figure 1).

Laparoscopic and robot-assisted pancreas left resection were both considered a minimally invasive left pancreatectomy (MILP). Factors like tumor size, tumor stage, the administration of neoadjuvant chemotherapy, and resection margins were considered in relation to the surgical approach (Table 1). Resections were divided by the approach used: open left pancreatectomy (OLP), laparoscopic left pancreatectomy (LLP), and robotic left pancreatectomy (RLP).

### 2.2. Institutional Review Board Statement

The study was conducted according to the guidelines of the Declaration of Helsinki, as each patient underwent informed consent prior to surgery. No institutional review board approval is needed for retrospective studies of already approved surgical procedures at the centers involved in this study.

### 2.3. Statistical Analysis

Time-to-event data were analyzed using Kaplan–Meier methods for surgical approaches, measuring the survival as the time from the date of pancreatic tumor diagnosis to the date of death. Patients who were lost to follow-up were censored on the date on which they were last known to be alive. Log-rank tests were used to compare survival curves.

Univariable Cox regression analyses were conducted to assess associations between clinical or pathological variables and OS, with age stratified as <65 vs. ≥65 years and tumor stage as <3 vs. ≥3. Given the relatively small sample size and the presence of multiple potential confounders, we employed a propensity score adjustment rather than a full multivariable Cox regression model.

Propensity scores were estimated using a logistic regression based on the following clinically relevant covariates: age, BMI, ASA (American Society of Anesthesiologists) score, gender, tumor stage, tumor diameter, lymph node status (positive vs. negative), resection margin status (R0 vs. R1), and neoadjuvant chemotherapy administration. The resulting propensity score was incorporated as a covariate in the final Cox regression model to adjust for potential baseline imbalances between surgical groups.

Statistical analysis was conducted using Microsoft Excel 2016 (Microsoft Corp., Albuquerque, NM, USA) and SPSS Statistics version 29.0.2.0 (IBM Corp., Armonk, NY, USA). Categorical variables are reported as absolute frequencies (*n*) and percentages (%), with comparisons made using Pearson’s chi-square test or Fisher’s exact test, depending on expected cell counts. Continuous variables are presented as mean ± standard deviation (SD), and group differences were evaluated using the Kruskal–Wallis test due to the non-normal distribution of several parameters. A *p*-value < 0.05 was considered statistically significant.

## 3. Results

### 3.1. Patient Characteristics

A total of 71 patients who underwent a left pancreatectomy for PDAC were analyzed. Of these, 13 patients underwent open surgery (18.3%), 42 patients underwent laparoscopic surgery (59.2%), and 16 patients underwent robotic surgery (22.5%) (Table 1).

The mean age of the total cohort was 68.92 ± 9.47 years, and 54.9% (*n* = 39/71) were female. The overall mean BMI was 26.45 ± 4.14 kg/m^2^. Most patients were classified as the ASA (American Society of Anesthesiologists) physical status class 2 (63.4%), and only two patients (2.9%) received neoadjuvant chemotherapy. The mean diameter of the largest resected tumor was 35.61 ± 22.23 mm, and the majority of tumors were classified as stage II (60.9%). The average number of lymph nodes retrieved was 16.1 ± 11.93, with 2.53 ± 9.5 pathologically positive nodes. An R0 resection was achieved in 69% of the cohort (Table 1).

### 3.2. Survival Analysis

**Figure 2:**
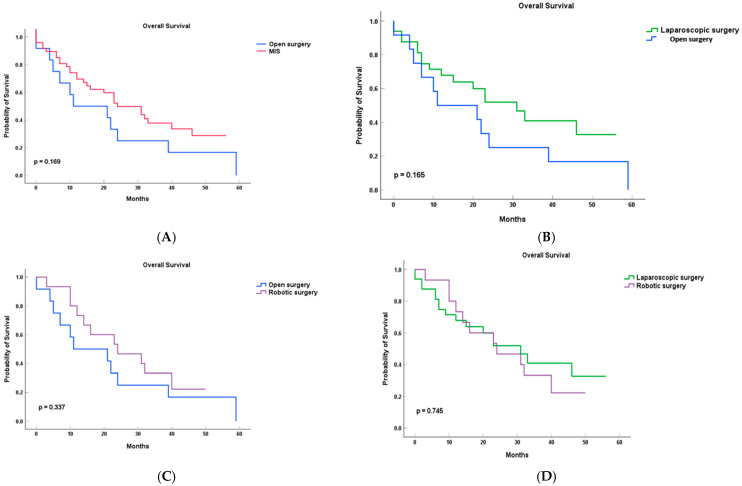
Overall Survival (OS) in patients with PDAC after left pancreatectomy, stratified by surgical approach. (**A**) Open (OLP) vs. minimally invasive (MILP); (**B**) laparoscopic (LLP) vs. open (OLP); (**C**) robotic (RLP) vs. open (OLP); and (**D**) laparoscopic (LLP) vs. robotic (RLP). Survival probabilities were estimated using Kaplan–Meier methods; comparisons were performed using log-rank tests.

Median overall survival was 11.0 months (95% CI: 0.0–29.67) in the open surgery group compared to 24.0 months (95% CI: 13.35–34.48) in the combined minimally invasive group, though this difference did not reach statistical significance (log-rank *p* = 0.169). Pairwise comparisons showed a longer median survival in the laparoscopic (24 vs. 11 months, *p* = 0.165) and robotic (24 vs. 11 months, *p* = 0.337) groups compared to open surgery; however, these differences were not statistically significant and should be interpreted with caution due to the limited sample size. When comparing individual minimally invasive approaches, the median OS was 24.0 months (95% CI: 13.53–34.48) for laparoscopic surgery and 24.0 months (95% CI: 5.07–42.94) for robotic surgery, with no significant difference (*p* = 0.745) between groups (Figure 2).

**Figure 3:**
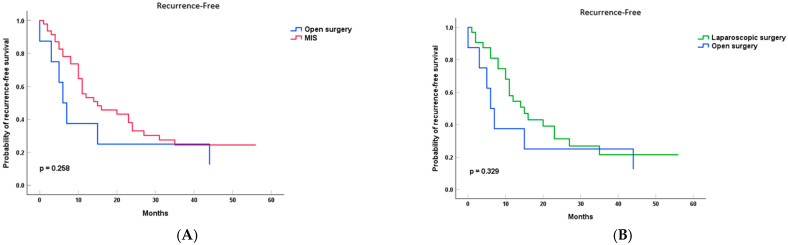

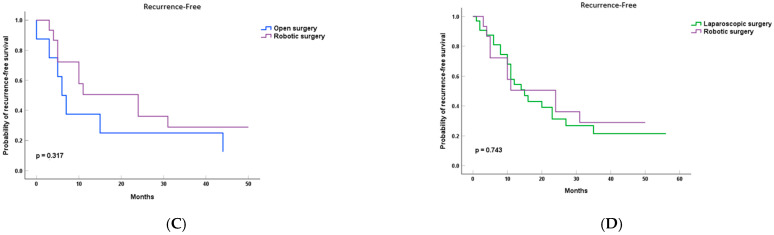
Recurrence-Free Survival (RFS) in patients with PDAC after left pancreatectomy, stratified by surgical approach. (**A**) Open (OLP) vs. minimally invasive (MILP); (**B**) laparoscopic (LLP) vs. open (OLP); (**C**) robotic (RLP) vs. open (OLP); and (**D**) laparoscopic (LLP) vs. robotic (RLP). Recurrence-free survival was estimated using Kaplan–Meier curves, and differences between groups were assessed using the log-rank test.

The median recurrence-free survival (RFS) was 6 months (95% CI: 3.23–8.77) in the open surgery group, compared to 15 months (95% CI: 4.2–25.8) in the minimally invasive surgery (MILP) group. This difference was not statistically significant (*p* = 0.258) (Figure 3).

When analyzed separately, the laparoscopic group had a median recurrence-free survival (RFS) of 15 months (95% CI: 4.2–25.8), while the robotic group had a median RFS of 24 months (95% CI: 7.71–40.29). However, none of the pairwise comparisons reached statistical significance (LLP vs. RLP: *p* = 0.743; LLP vs. OLP: *p* = 0.329; and RLP vs. OLP: *p* = 0.317). These differences should be interpreted with caution given the small subgroup sizes and limited statistical power.

### 3.3. Univariable Covariant Balance and Propensity-Adjusted Analysis

In the univariable Cox regression, several covariates were significantly associated with overall survival. A tumor stage ≥ 3 was associated with worse survival (HR = 3.250, 95% CI [1.501–7.037], and *p* = 0.003). A higher BMI was also associated with poorer outcomes (HR = 1.113, 95% CI [1.025–1.209], and *p* = 0.011), as was pathologic lymph node positivity (HR = 1.154, 95% CI [1.022–1.303], and *p* = 0.021).

The propensity score covariate itself was also not significantly associated with survival (HR = 0.884, *p* = 0.880) (Table 2 and Table 3), with no statistically significant differences observed between the two groups across all baseline covariates before and after propensity score adjustment, indicating adequate balance (Table 4).

The surgical approach (open vs. minimally invasive surgery [MIS]) showed no statistically significant association with overall survival (HR = 0.608, *p* = 0.178). After a propensity score adjustment, this association remained non-significant with regard to the overall survival (HR = 0.759, *p* = 0.595) (Table 3).

## 4. Discussion

Over the past decade, the minimally invasive left pancreatectomy (MILP), including both laparoscopic and robotic techniques, has seen significant advancements [3,23]. Continuous development in surgical methods and technological innovations have led to shorter hospital stays, reduced complication rates, less blood loss, and improved postoperative quality of life, all without increasing the cost [9,12,24]. These benefits have been recognized in international guidelines, including the Miami 2020 and Brescia 2023 recommendations and the LEOPARD prospective randomized trial [3,25,26].

However, these studies have primarily focused on short-term outcomes in heterogenous patient cohorts [3,26]. Following the validation of the minimally invasive approach in malignant cases, more recent studies have shifted their focus towards investigating oncological outcomes, such as lymph nsode harvest and resection margins, particularly in the minimally invasive setting compared to the open approach [14,26]. While some evidence suggests that the robotic approach may be associated with a lower incidence of postoperative pancreatic fistula (POPF) compared to the laparoscopic technique, major morbidity, mortality, and oncological efficacy appears similar across all three surgical modalities [12]. However, factors such as tumor size, location, and involvement of adjacent organs or vessels still have a major influence in the selection of surgical approach [27]. Hence, the availability of different surgical approaches may aid in selecting the appropriate procedure without compromising the oncological outcome, as improving overall survival remains a critical goal.

The purpose of the current study (SIMPLR-2) is to investigate long-term oncological outcomes according to different surgical approaches: open left pancreatectomy (OLP), laparoscopic left pancreatectomy (LLP), and robotic left pancreatectomy (RLP) in PDAC. By excluding other malignancies, we aimed to provide a specific analysis of the oncologic effectiveness of MILP for pancreatic adenocarcinoma, the most prevalent pancreatic cancer.

In our cohort, minimally invasive approaches were associated with a trend toward an improved overall survival and recurrence-free survival compared to open surgery, a median OS in the MILP group (24.0 months) compared to the ODP group (11.0 months; *p* = 0.169), and a median RFS (15.0 vs. 6.0 months; these differences did not reach statistical significance, *p* = 0.258). Comparable findings were reported by Jin et al. and Nassour et al., who observed no significant difference in OS but did demonstrate a statistically significant improvement in RFS with MILP. This benefit can be explained by the improved postoperative short-term outcomes (less POPF and shorter hospital stay), hence the timely initiation of adjuvant therapy [27,28]. Although minimally invasive approaches in our cohort were associated with a longer median survival, these differences did not reach statistical significance. Given the limited sample size and exploratory design, no definitive conclusions can be drawn, and larger prospective trials are required to validate these findings. Nonetheless, the fact that only two patients in this cohort received neoadjuvant treatments begs the question as to whether neoadjuvant treatments should be advocated for more patients with resectable left-sided pancreatic cancer, particularly in patients undergoing open resection.

Until recently, LLP and RLP approaches were often grouped together under the umbrella of the minimally invasive approach when compared to open surgery [26,29]. However, laparoscopic and robotic approaches differ significantly in operative technique, cost, and learning curve, which may influence perioperative and oncological outcomes [20]. Hence, it is important to assess them separately in order to fully understand their benefits and challenges. Following the first meta-analysis by Zhou et al. in 2016, which involved 568 patients and compared LLP and RLP, a number of recent studies have explored the outcomes of these two approaches [26,27].

Our analysis included a subgroup analysis to understand the specific contribution of each minimally invasive technique, comparing not only the two minimally invasive approaches, but also each minimally invasive technique, which were individually compared to the open approach. The results showed a comparable OS and RFS between laparoscopic and robotic approaches also when compared individually to the open approach (LLP vs. RLP: *p* = 0.743; LLP vs. OLP: *p* = 0.329; and RLP vs. OLP: *p* = 0.317). Although, the robotic group demonstrated a numerically longer survival in both outcomes; however, none of the pairwise comparisons reached statistical significance. Regarding the comparison between LLP and RLP, our findings aligned with the largest retrospective analysis from Korea, conducted by Hong et al., analyzing 88 PDAC patients (12 RLP, 76 LLP), and found no significant difference in median overall survival (OS) or disease-free survival (DFS) [30]. Similarly, Qu et al. examined survival outcomes in 70 PDAC patients (35 RLP, 35 LLP) using propensity score matching and reported no significant differences in OS or DFS [31].

Tumor stage and lymph node involvement have also been identified in previous studies as prognostic factors for overall survival [6,7,9,17]. In our cohort, after univariable Cox regression analysis, several clinical factors correlate significantly with overall survival in patients with PDAC, including tumor size (≥3, *p* = 0.003), higher BMI (*p* = 0.011), and lymph node positivity (*p* = 0.021). However, the surgical approach (open vs. minimally invasive) showed no statistically significant association with overall survival.

After propensity score adjustment, the association between surgical approach and survival remained non-significant, and the propensity score covariate itself was not independently associated with survival. These findings may suggest that oncologic outcomes in PDAC are more likely influenced by tumor biology than by the choice of surgical technique. Additionally, the resection margin status is widely recognized as a crucial oncological factor [3,12]; our study showed comparable R0 resection rates across approaches with the highest rate observed in the robotic group (95.2%), followed by laparoscopic (75%) and open (70%). These findings were supported by the DIPLOMA randomized control trial, which confirmed the oncological safety of MILP by demonstrating non-inferiority when compared to open surgery [26]. However, this trial included—but was not exclusive to—patients with PDAC and did not compare RLP vs. LLP directly.

Additionally, a potential source of bias in the interpretation of outcomes in minimally invasive pancreatic surgery lies in the selection of patients with more favorable risk profiles for laparoscopic or robotic approaches. This selection bias may substantially affect the association between surgical technique and postoperative outcomes. This issue has been addressed in the recent literature. In their network meta-analysis, Joseph et al. pointed out that, despite generally favorable outcomes for laparoscopic pancreatoduodenectomy (LPD), the included randomized trials were often underpowered and could not exclude the possibility of selection bias [32]. Differences in conversion rates and surgeon experience across the studies further underscore the need for cautious interpretation [32]. In the case of robotic procedures (RPD), notable variations in surgeon experience have been observed, complicating the interpretation of results and highlighting the impact of the learning curve, which is characterized by heterogeneous definitions and is inconsistently accounted for across trials [32]. Future studies should therefore not only be randomized and controlled but also incorporate standardized risk adjustment and patient-centered outcomes to reliably determine the true benefit of minimally invasive approaches.

Overall, cumulative data suggest that MILP, including both RLP and LLP, may offer a better oncologic outcome in selected cases compared to OLP. Furthermore, RLP could provide additional benefits due to enhanced visualization and tremor reduction, leading to greater surgical precision [3,18]. However, the absence of prospective, multicenter trials with longer follow-ups are still lacking and remains a limitation, and no randomized controlled trials (RCTs) have yet to compare long-term survival outcomes across the three different surgical approaches for PDAC.

While our study does not introduce novel statistical methodologies or a randomized design, it represents the first international multicenter effort to directly compare open, laparoscopic, and robotic approaches for PDAC in terms of long-term survival. By focusing exclusively on histologically confirmed PDAC cases and applying a propensity score adjustment across high-volume expert centers, we aimed to reduce selection bias and increase disease-specific validity. However, our findings should be considered exploratory, and future randomized studies are required to confirm these trends.

A key limitation is the small number of patients in the robotic subgroup (*n* = 16), resulting in wide confidence intervals and limited statistical robustness for this group. Therefore, any potential benefits of robotic surgery observed in this study must be interpreted with caution.

While all participating institutions are recognized high-volume centers with established expertise in pancreatic surgery, detailed documentation of surgical volume and individual surgeon experience was beyond the scope of this retrospective analysis. Additionally, no uniform training protocol was implemented across centers. These aspects may have contributed to the variability in outcomes and should be addressed in future prospective studies with standardized procedural frameworks.

Another limitation lies in the retrospective, multicenter design, which may introduce heterogeneity and information bias. However, the primary outcome of this study—overall survival—was obtained from official population registries, ensuring a reliable follow-up. Furthermore, the study was not powered to detect small-to-moderate differences between surgical subgroups; accordingly, all subgroup comparisons should be interpreted conservatively.

## 5. Conclusions

To date and to our knowledge, a large-scale international comparison of OLP, RLP, and LLP in high-volume centers for resectable pancreatic cancer is still lacking.

European guidelines emphasize that hospital volume and surgeon expertise significantly impact patient outcomes, making the choice of surgical approach dependent on the patient and tumor characteristics as well as the proficiency of the surgical team, which are all considered as crucial factors for OS and RFS.

Additionally, as RLP adoption increases, evaluating differences within MILP approaches regarding safety and efficacy is essential, with further research needed to determine long-term survival benefits.

## Figures and Tables

**Table 1:** Demographic data in patients with PDAC.

	Total	Open	Laparoscopic	Robotic	*p*-Value
	(*n* = 71)	(*n* = 13)	(*n* = 42)	(*n* = 16)	
**Gender**					
Female	39 (54.9%)	6 (46.2%)	23 (54.8%)	10 (62.5%)	0.713
Male	32 (45.1%)	7 (53.8%)	19 (45.2%)	6 (37.5%)	
**Age**					
Mean (SD)	68.92 (9.47)	63.62 (11.77)	70.52 (7.39)	69 (11.29)	0.025
<65	19(26.8%)	7 (53.8%)	7 (16.7%)	5 (31.3%)	
≥65	52 (73.2%)	6 (46.2%)	35 (83.3%)	11 (68.8%)	
**BMI**					
Mean (SD)	26.45 (4.14)	27.37 (3.89)	27.29 (3.8)	23.65 (4.13)	0.006
**ASA Score**					
1	0		0	0	0.382
2	45 (63.4%)	8 (61.5%)	29 (69%)	8 (50%)	
3	26 (36.6%)	5 (38.5%)	13 (31%)	8 (50%)	
4	0		1 (1.3%)	0	
**Neoadjuvant** **Chemotherapy**					
No	68 (97.1%)	12 (92.3%)	40 (97.6%)	16 (100%)	0.389
Yes	2 (2.9%)	1 (7.7%)	1 (2.4%)	0 (0%)	
**Diameter of** **Largest Resected** **Tumor (mm)**					
Mean (SD)	35.61 (22.23)	57.46 (23.6)	39.9 (15.9)	25.64 (11.41)	<0.001
**Stage**					
0	1 (1.4%)		1 (2.4%)	0	<0.001
1	8 (11.6%)	2 (16.7%)	1 (2.4%)	5 (31.3%)	
2	42 (60.9%)	9 (75.0%)	29 (70.7%)	4 (25%)	
3	12 (17.4%)	1 (8.3%)	4 (9.08%)	7 (43.8%)	
4	6 (8.7%)		6 (14.6%)	0	
**# LNN Retrieved**					
Mean (SD)	16.1 (11.93)	23.08 (14.48)	14.51 (8.32)	27.81 (10.63)	<0.001
**# Pathologic.LNN**					
Mean (SD)	2.53 (9.5)	2.08 (2.63)	1.56 (1.42)	2.94 (3.09)	0.474
**Resection margin**					
R0	49 (69%)	9 (69.2%)	25 (59.5%)	15 (93.8%)	0.034
R1	22 (31%)	4 (30.8%)	17 (40.5%)	1 (6.3%)	

**Table 2:** Univariate analysis in patients with PDAC.

		HR (95% CI)	*p*-Value
Gender	0.848	(0.446–1.609)	0.613
Age (<65 vs. ≥65)	0.767	(0.392–1.502)	0.439
BMI	1.113	(1.025–1.209)	0.011
ASA-Score (2 or 3)	1.395	(0.724–2.688)	0.319
Stage (<3 vs. ≥3)	3.250	(1.501–7.037)	0.003
Resection margin (R0 vs. R1)	1.031	(0.500–2.126)	0.934
Surgical approach (OS vs. MIS)	0.608	(0.295–1.254)	0.178
Diameter of largest resected tumor (mm)	1.002	(0.987–1.017)	0.809
Neoadjuvant chemotherapy	0.858	(0.117–6.289)	0.880
# LNN Retrieved	1.003	(0.976–1.031)	0.823
# Pathologic LNN	1.154	(1.022–1.303)	0.021
Resection margin	1.031	(0.500–2.126)	0.934

**Table 3:** Multivariable analysis in patients with PDAC.

	HR (95% CI)		*p*-Value
**OS vs. MIS**	0.759	(0.274–2.102)	0.595
Propensity score	0.884	(0.179–4.365)	0.880

**Table 4:** Covariate balance.

Covariate	Open (Mean (±SD) or *n* (%))	MIS (Mean (±SD) or *n* (%))	*p*-Value (Bevor PS Adjustment)	*p*-Value (After PS Adjustment)
Gender (male)	7 (21.9%)	25 (78.1%)	0.255	0.634
Age ≥ 65	6 (11.5%)	46 (88.5%)	0.015	0.263
BMI	27.37 (±3.89)	26.23 (±4.21)	0.509	0.228
ASA (1 or 2)	8 (17.8%)	37 (82.2%)	0.886	0.184
Neoadjuvant Chemotherapy (no)	12 (17.6%)	56 (82.4%)	0.307	0.718
Diameter of Largest Resected Tumor (mm)	57.46 (±23.6)	36.27 (±16.06)	<0.001	0.19
Stage (<3)	11 (21.6%)	40 (78.4%)	0.067	0.077
# LNN Retrieved	23.08 (±14.48)	18.69 (±10.95)	0.267	0.163
# Pathologic.LNN	2.08 (±2.63)	2 ± 2.17	0.583	0.844
Resection Margin (R0)	9 (18.4%)	40 (81.6%)	0.765	0.847

## Data Availability

Data available upon reasonable request.
